# KMg_0.09_Fe_1.91_(PO_4_)_2_

**DOI:** 10.1107/S1600536812023975

**Published:** 2012-05-31

**Authors:** Michael M. Yatskin, Igor V. Zatovsky, Vyacheslav N. Baumer, Ivan V. Ogorodnyk, Nikolay S. Slobodyanik

**Affiliations:** a Department of Inorganic Chemistry, Taras Shevchenko National University, 64, Volodymyrska Str., 01601, Kyiv, Ukraine; b STC "Institute for Single Crystals", NAS of Ukraine, 60 Lenin Ave., 61001, Kharkiv, Ukraine

## Abstract

KMg_0.09_Fe_1.91_(PO_4_)_2_, potassium [iron(II)/magnesium] iron(III) bis(orthophosphate), is a solid solution derived from compounds with general formula K*M* ^II^Fe(PO_4_)_2_ (*M* ^II^ = Fe, Cu), in which the Mg atoms substitute Fe atoms only in the octa­hedrally surrounded sites. The framework of the structure is built up from [FeO_5_] trigonal bipyramids and [*M*O_6_] (*M* = (Fe, Mg) octa­hedra sharing corners and edges and connected by two types of bridging PO_4_ tetra­hedra. The K^+^ cations are nine-coordinated and are situated in channels running along [101].

## Related literature

For the structure of KFe_2_(PO_4_)_2_, see: Yakubovich *et al.* (1986 ▶) and for the structure of KCuFe(PO_4_)_2_, see: Badri *et al.* (2011 ▶). For calculations of bond-valence sums, see: Brown & Altermatt (1985 ▶).

## Experimental

### 

#### Crystal data

KMg_0.09_Fe_1.91_(PO_4_)_2_
*M* *_r_* = 337.97
Monoclinic, 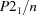

*a* = 7.8444 (3) Å
*b* = 10.0033 (3) Å
*c* = 9.0371 (4) Å
β = 114.838 (5)°
*V* = 643.54 (4) Å^3^
*Z* = 4
Mo *K*α radiation
μ = 5.48 mm^−1^
*T* = 293 K
0.12 × 0.02 × 0.02 mm

#### Data collection

Oxford Diffraction XCalibur-3 CCD diffractometer
Absorption correction: multi-scan (Blessing, 1995 ▶) *T*_min_ = 0.857, *T*_max_ = 0.903
10004 measured reflections
2230 independent reflections
2155 reflections with *I* > 2σ(*I*)
*R*_int_ = 0.034

#### Refinement

*R*[*F* ^2^ > 2σ(*F* ^2^)] = 0.042
*wR*(*F* ^2^) = 0.117
*S* = 1.19
2230 reflections
120 parameters
Δρ_max_ = 2.08 e Å^−3^
Δρ_min_ = −1.01 e Å^−3^

### 

Data collection: *CrysAlis CCD* (Oxford Diffraction, 2006 ▶); cell refinement: *CrysAlis CCD*; data reduction: *CrysAlis RED* (Oxford Diffraction, 2006 ▶); program(s) used to solve structure: *SHELXS97* (Sheldrick, 2008 ▶); program(s) used to refine structure: *SHELXL97* (Sheldrick, 2008 ▶); molecular graphics: *DIAMOND* (Brandenburg, 1999 ▶); software used to prepare material for publication: *WinGX* publication routines (Farrugia, 1999 ▶) and *enCIFer* (Allen *et al.*, 2004 ▶)’.

## Supplementary Material

Crystal structure: contains datablock(s) global, I. DOI: 10.1107/S1600536812023975/wm2629sup1.cif

Structure factors: contains datablock(s) I. DOI: 10.1107/S1600536812023975/wm2629Isup2.hkl

Additional supplementary materials:  crystallographic information; 3D view; checkCIF report

## Figures and Tables

**Table 1:** Selected bond lengths (Å)

Fe1—O11	1.955 (3)
Fe1—O24	1.984 (3)
Fe1—O24^i^	1.989 (3)
Fe1—O13	2.041 (3)
Fe1—O12	2.060 (3)
(Fe,Mg)2—O22^ii^	1.947 (3)
(Fe,Mg)2—O21^iii^	1.959 (3)
(Fe,Mg)2—O23	1.971 (3)
(Fe,Mg)2—O14	2.003 (3)
(Fe,Mg)2—O13^iii^	2.072 (3)
(Fe,Mg)2—O12^iv^	2.133 (3)

Symmetry codes: (i) 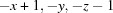; (ii) 
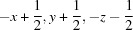
; (iii) 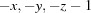; (iv) 
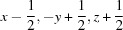
.

**Fig. 1.**
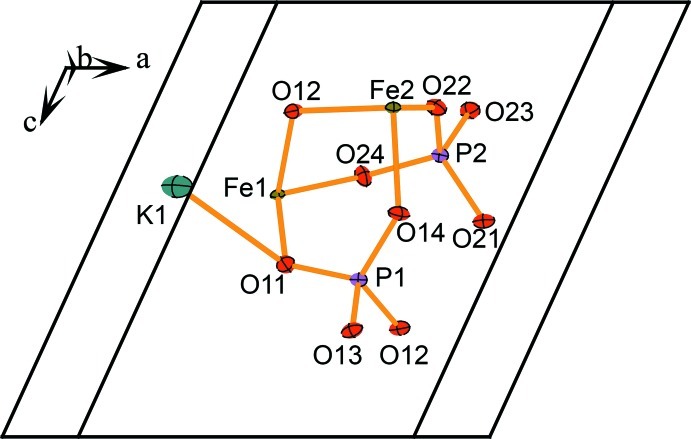
The asymmetric unit of KMg0.09Fe1.91(PO4)2, showing displacement ellipsoids at the 50% probability level.

**Fig. 2.**
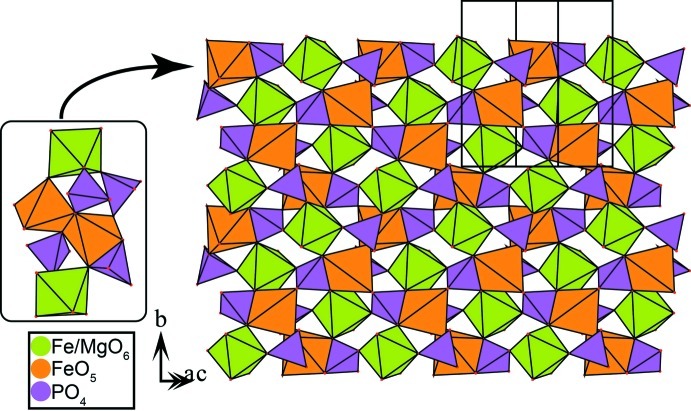
Elementary fragments in the titled compound.

**Fig. 3.**
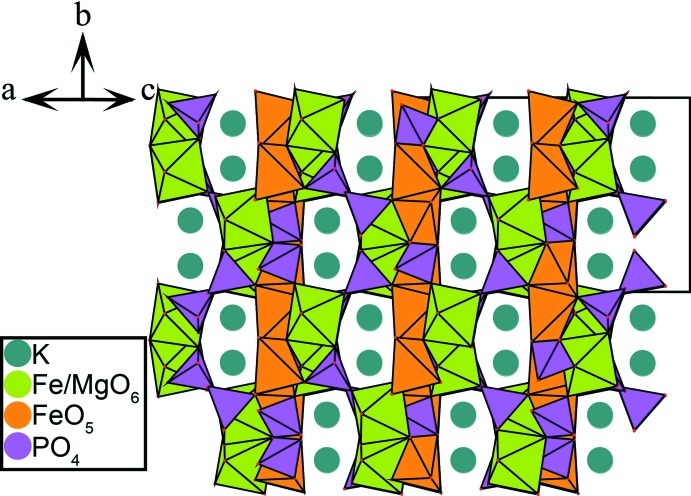
A projection of the structure of KMg0.09Fe1.91(PO4)2 along [101].

## supplementary crystallographic information

### Comment

KMg_0.09_Fe_1.91_(PO_4_)_2_ is a solid solution and crystallizes in the KFe_2_(PO_4_)_2_ structure type (originally reported in space group *P*2_1_/*a*; Yakubovich *et al.*, 1986). KCuFe(PO_4_)_2_ (originally reported in space group *P*2_1_/*n*; Badri *et al.*, 2011) is another isotypic member.

The asymmetric unit of KMg_0.09_Fe_1.91_(PO_4_)_2_ consist of one K, two Fe (one is partially occupied by Mg), two P and eight oxygen positions (Fig. 1). The main building block involves two [(*M*)O_6_] octahedra (*M* = Fe2,Mg) and two [Fe1O_5_] trigonal bipyramids connected by four orthophosphate tetrahedra. Such blocks are aggregated into a three-dimensional framework which can be described by the general formula [Mg_0.09_Fe_1.91_(PO_4_)_2_]_∞_^-^ (Fig. 2). It should be noted, that Mg was determined only in the six-coordinated position while the five-coordinated position is occupied only by Fe.

The *M* sites lie in a rather distorted octahedron which vertices are shared by two types of orthophosphate tetrahedra (the Fe2—O distances varies from 1.947 (3) to 2.133 (3) Å). Completeness of the Fe1 environment is achieved by three orthophosphate tetrahedra connected to the metal atom only by one vertex and one orthophosphate tetrahedron connected *via* an edge (the Fe1—O distances lie in the range 1.955 (3) to 2.060 (3) Å). In comparison with KCuFe(PO_4_)_2_ (Badri *et al.*, 2011), the bond lengths of Fe2—O are very close in both structures. In KCuFe(PO_4_)_2_ this position is occupied by Fe^3+^, whereas in the structure of KMg_0.09_Fe_1.91_(PO_4_)_2_ it is occupied by both Mg and Fe. The average Fe—O lengths of both positions are very close (Fe1—O_average_ = 2,00 Å; Fe2—O_average_ = 2,01 Å). Thus it can be assumed that Fe^2+^ and Fe^3+^ are distributed over both positions which is confirmed by bond valence sums (BVS) calculations (Brown & Altermatt, 1985). For both positions the BVS values were found inbetween those expected for full occupancy with Fe^2+^ and Fe^3+^. Fe1: 2.6 valence units (v.u.) with the parameters of Fe^3+^ and 2.4 v.u. with the parameters of Fe^2+^; Fe2: 3.0 v.u. with the parameters of Fe^3+^ and 2.5 v.u. with the parameters of Fe^2+^.

The geometry of the orthophosphate tetrahedra is close to regular with P—O bond length ranging from 1.513 (3) to 1.567 (3) Å. The BVS values for both P atoms are close to the expected 5 (4.91 v.u. for P1; 4.95 v.u. for P2).

The potassium atoms are located in hexagonal channels running along [101] (Fig. 3). The distorted [KO_9_] coordination polyhedron is formed by nine phosphate O atoms assuming a cut-off distance of 3.2 Å.

### Experimental

The title compound was obtained from high-temperature solution in the pseudo-system K_2_O—P_2_O_5_—MoO_3_—Fe_2_O_3_—MgO. The calculated amounts of KPO_3_ (3.54 g), H_3_PO_4_ (0.98 g), Fe_2_O_3_ (0.96 g), MgO (0.48 g) and K_2_Mo_2_O_7_ (1.136 g) in molar ratios of K/P/Fe/Mg/Mo equal to 1:1.1:0.3:0.3:0.15 was mixed, ground in an agate mortar and heated up to 1273 K in a platinum crucible. Then the temperature was cooled down to 873 K during 8 h (at a constant rate) to crystallize the desired crystals. The flux was poured out of the crucible and the obtained crystals were recovered from the remaining solidified flux using hot water.

### Refinement

The atomic positions and labelling of atoms are the same as in Badri *et al.* (2011) to simplify any comparison.

In the first steps of structure refinement, the Mg atoms were placed into the same positions as Fe. The coordinates and the ADPs of both Mg1 and Fe1, Mg2 and Fe2 sites were constrained to be equal. The corresponding occupancies were freely refined but constrained to unity. It was found that the Mg occupancy in the *M*1 position is negative. Thus the occupancy of Fe in this site was set to 1. The Mg quantity was refined only in the position *M*2. At the same time, we refined the occupancy of K1 site freely; it was found to be 1.

The remaining highest positive electron density was found at a distance of 0.69 Å from Fe1 and the highest negative density at 0.44 Å from P1.

### Crystal data

**Table:**

KMg_0.09_Fe_1.91_(PO_4_)_2_	*F*(000) = 654.4
*M**_r_* = 337.97	*D*_x_ = 3.488 Mg m^−^^3^
Monoclinic, *P*2_1_/*n*	Mo *K*α radiation, λ = 0.71073 Å
Hall symbol: -P 2yn	Cell parameters from 10004 reflections
*a* = 7.8444 (3) Å	θ = 2.9–32°
*b* = 10.0033 (3) Å	µ = 5.48 mm^−^^1^
*c* = 9.0371 (4) Å	*T* = 293 K
β = 114.838 (5)°	Needle, light pink
*V* = 643.54 (4) Å^3^	0.12 × 0.02 × 0.02 mm
*Z* = 4	

### Data collection

**Table:**

Oxford Diffraction XCalibur-3 CCD diffractometer	2230 independent reflections
Radiation source: fine-focus sealed tube	2155 reflections with *I* > 2σ(*I*)
Graphite monochromator	*R*_int_ = 0.034
φ and ω scans	θ_max_ = 32°, θ_min_ = 2.9°
Absorption correction: multi-scan (Blessing, 1995)	*h* = −10→11
*T*_min_ = 0.857, *T*_max_ = 0.903	*k* = −14→14
10004 measured reflections	*l* = −13→13

### Refinement

**Table:**

Refinement on *F*^2^	Primary atom site location: structure-invariant direct methods
Least-squares matrix: full	Secondary atom site location: difference Fourier map
*R*[*F*^2^ > 2σ(*F*^2^)] = 0.042	*w* = 1/[σ^2^(*F*_o_^2^) + (0.0371*P*)^2^ + 6.6825*P*] where *P* = (*F*_o_^2^ + 2*F*_c_^2^)/3
*wR*(*F*^2^) = 0.117	(Δ/σ)_max_ < 0.001
*S* = 1.19	Δρ_max_ = 2.08 e Å^−^^3^
2230 reflections	Δρ_min_ = −1.01 e Å^−^^3^
120 parameters	Extinction correction: *SHELXL*
0 restraints	Extinction coefficient: 0.0145 (14)

### Special details

**Table:**

Geometry. All e.s.d.'s (except the e.s.d. in the dihedral angle between two l.s. planes) are estimated using the full covariance matrix. The cell e.s.d.'s are taken into account individually in the estimation of e.s.d.'s in distances, angles and torsion angles; correlations between e.s.d.'s in cell parameters are only used when they are defined by crystal symmetry. An approximate (isotropic) treatment of cell e.s.d.'s is used for estimating e.s.d.'s involving l.s. planes.
Refinement. Refinement of *F*^2^ against ALL reflections. The weighted *R*-factor *wR* and goodness of fit *S* are based on *F*^2^, conventional *R*-factors *R* are based on *F*, with *F* set to zero for negative *F*^2^. The threshold expression of *F*^2^ > σ(*F*^2^) is used only for calculating *R*-factors(gt) *etc*. and is not relevant to the choice of reflections for refinement. *R*-factors based on *F*^2^ are statistically about twice as large as those based on *F*, and *R*- factors based on ALL data will be even larger.

### Fractional atomic coordinates and isotropic or equivalent isotropic displacement parameters (Å^2^)

**Table:**

	*x*	*y*	*z*	*U*_iso_*/*U*_eq_	Occ. (<1)
K1	0.42060 (18)	−0.13394 (13)	0.07587 (14)	0.0267 (3)	
Fe1	0.37733 (7)	0.11765 (5)	−0.55702 (6)	0.00590 (15)	
Fe2	0.01350 (8)	0.12386 (5)	−0.25744 (7)	0.0065 (2)	0.912 (8)
Mg2	0.01350 (8)	0.12386 (5)	−0.25744 (7)	0.0065 (2)	0.088 (8)
P1	0.12822 (14)	0.16101 (10)	−0.86090 (12)	0.0088 (2)	
P2	0.26703 (14)	−0.09247 (10)	−0.35182 (12)	0.0085 (2)	
O11	0.4483 (4)	0.2619 (3)	−0.3964 (4)	0.0127 (5)	
O12	0.2987 (4)	0.2495 (3)	−0.7494 (4)	0.0112 (5)	
O13	0.1465 (4)	0.0404 (3)	−0.7444 (4)	0.0120 (5)	
O14	0.1437 (5)	0.1137 (3)	−0.0138 (4)	0.0138 (6)	
O21	0.0945 (4)	−0.1311 (3)	−0.5040 (4)	0.0133 (5)	
O22	0.3516 (4)	−0.2128 (3)	−0.2422 (4)	0.0126 (5)	
O23	0.2247 (4)	0.0124 (3)	−0.2489 (4)	0.0125 (5)	
O24	0.4195 (4)	−0.0311 (3)	−0.4003 (4)	0.0131 (5)	

### Atomic displacement parameters (Å^2^)

**Table:**

	*U*^11^	*U*^22^	*U*^33^	*U*^12^	*U*^13^	*U*^23^
K1	0.0313 (6)	0.0285 (6)	0.0181 (5)	−0.0129 (4)	0.0080 (4)	−0.0006 (4)
Fe1	0.0070 (2)	0.0052 (2)	0.0045 (2)	−0.00011 (16)	0.00150 (18)	0.00047 (16)
Fe2	0.0080 (3)	0.0057 (3)	0.0054 (3)	−0.00005 (17)	0.0023 (2)	−0.00025 (17)
Mg2	0.0080 (3)	0.0057 (3)	0.0054 (3)	−0.00005 (17)	0.0023 (2)	−0.00025 (17)
P1	0.0103 (4)	0.0086 (4)	0.0068 (4)	−0.0005 (3)	0.0030 (3)	0.0000 (3)
P2	0.0100 (4)	0.0082 (4)	0.0074 (4)	0.0006 (3)	0.0036 (3)	0.0001 (3)
O11	0.0136 (12)	0.0126 (13)	0.0120 (12)	−0.0031 (10)	0.0054 (10)	−0.0031 (10)
O12	0.0127 (12)	0.0110 (12)	0.0077 (11)	−0.0028 (10)	0.0020 (10)	0.0005 (9)
O13	0.0128 (12)	0.0104 (12)	0.0095 (12)	−0.0022 (10)	0.0015 (10)	0.0019 (10)
O14	0.0176 (14)	0.0159 (14)	0.0076 (12)	−0.0002 (11)	0.0050 (11)	−0.0024 (10)
O21	0.0151 (13)	0.0150 (13)	0.0073 (12)	0.0000 (10)	0.0024 (10)	−0.0011 (10)
O22	0.0157 (13)	0.0117 (13)	0.0113 (12)	0.0041 (10)	0.0066 (11)	0.0026 (10)
O23	0.0134 (12)	0.0120 (13)	0.0114 (12)	0.0022 (10)	0.0045 (10)	−0.0022 (10)
O24	0.0134 (12)	0.0123 (13)	0.0164 (13)	0.0033 (10)	0.0089 (11)	0.0049 (11)

### Geometric parameters (Å, º)

**Table:**

K1—O22	2.806 (3)	Fe2—O22^v^	1.947 (3)
K1—O23^i^	2.830 (3)	Fe2—O21^vi^	1.959 (3)
K1—O11^ii^	2.854 (3)	Fe2—O23	1.971 (3)
K1—O11^i^	2.930 (3)	Fe2—O14	2.003 (3)
K1—O21^iii^	2.955 (3)	Fe2—O13^vi^	2.072 (3)
K1—O12^ii^	3.007 (3)	Fe2—O12^vii^	2.133 (3)
K1—O23	3.054 (3)	P1—O14^viii^	1.513 (3)
K1—O24^i^	3.131 (3)	P1—O11^ix^	1.520 (3)
K1—O14	3.167 (3)	P1—O12	1.567 (3)
Fe1—O11	1.955 (3)	P1—O13	1.567 (3)
Fe1—O24	1.984 (3)	P2—O21	1.520 (3)
Fe1—O24^iv^	1.989 (3)	P2—O22	1.523 (3)
Fe1—O13	2.041 (3)	P2—O23	1.528 (3)
Fe1—O12	2.060 (3)	P2—O24	1.562 (3)
			
O22—K1—O23^i^	114.12 (10)	O23—Fe2—O13^vi^	93.03 (13)
O22—K1—O11^ii^	66.54 (9)	O14—Fe2—O13^vi^	88.97 (12)
O23^i^—K1—O11^ii^	175.96 (10)	O22^v^—Fe2—O12^vii^	86.54 (12)
O22—K1—O11^i^	136.60 (10)	O21^vi^—Fe2—O12^vii^	91.89 (12)
O23^i^—K1—O11^i^	77.66 (9)	O23—Fe2—O12^vii^	175.72 (12)
O11^ii^—K1—O11^i^	104.68 (8)	O14—Fe2—O12^vii^	92.19 (12)
O22—K1—O21^iii^	55.69 (9)	O13^vi^—Fe2—O12^vii^	88.90 (12)
O23^i^—K1—O21^iii^	91.67 (9)	O14^viii^—P1—O11^ix^	112.93 (18)
O11^ii^—K1—O21^iii^	91.87 (9)	O14^viii^—P1—O12	112.93 (17)
O11^i^—K1—O21^iii^	83.54 (9)	O11^ix^—P1—O12	108.38 (18)
O22—K1—O12^ii^	121.25 (9)	O14^viii^—P1—O13	110.67 (18)
O23^i^—K1—O12^ii^	121.56 (9)	O11^ix^—P1—O13	110.31 (17)
O11^ii^—K1—O12^ii^	59.29 (8)	O12—P1—O13	100.98 (16)
O11^i^—K1—O12^ii^	49.85 (8)	O21—P2—O22	111.52 (18)
O21^iii^—K1—O12^ii^	104.06 (9)	O21—P2—O23	112.74 (18)
O22—K1—O23	49.28 (9)	O22—P2—O23	107.06 (17)
O23^i^—K1—O23	107.82 (8)	O21—P2—O24	110.00 (18)
O11^ii^—K1—O23	69.39 (9)	O22—P2—O24	108.52 (17)
O11^i^—K1—O23	170.19 (9)	O23—P2—O24	106.79 (18)
O21^iii^—K1—O23	104.12 (9)	P1^x^—O11—Fe1	118.74 (18)
O12^ii^—K1—O23	121.31 (9)	P1^x^—O11—K1^v^	124.41 (17)
O22—K1—O24^i^	159.49 (9)	Fe1—O11—K1^v^	86.74 (11)
O23^i^—K1—O24^i^	48.85 (8)	P1^x^—O11—K1^i^	95.94 (14)
O11^ii^—K1—O24^i^	129.51 (9)	Fe1—O11—K1^i^	106.47 (13)
O11^i^—K1—O24^i^	57.90 (9)	K1^v^—O11—K1^i^	124.91 (11)
O21^iii^—K1—O24^i^	127.13 (9)	P1—O12—Fe1	93.03 (14)
O12^ii^—K1—O24^i^	78.93 (8)	P1—O12—Fe2^xi^	141.86 (18)
O23—K1—O24^i^	119.28 (9)	Fe1—O12—Fe2^xi^	116.63 (14)
O22—K1—O14	98.12 (9)	P1—O12—K1^v^	91.93 (13)
O23^i^—K1—O14	102.39 (9)	Fe1—O12—K1^v^	80.92 (10)
O11^ii^—K1—O14	73.59 (9)	Fe2^xi^—O12—K1^v^	114.88 (12)
O11^i^—K1—O14	120.87 (9)	P1—O13—Fe1	93.73 (14)
O21^iii^—K1—O14	153.74 (9)	P1—O13—Fe2^vi^	136.95 (18)
O12^ii^—K1—O14	87.46 (9)	Fe1—O13—Fe2^vi^	128.51 (15)
O23—K1—O14	50.59 (8)	P1^xii^—O14—Fe2	142.0 (2)
O24^i^—K1—O14	77.83 (8)	P1^xii^—O14—K1	108.69 (16)
O11—Fe1—O24	96.53 (13)	Fe2—O14—K1	107.41 (12)
O11—Fe1—O24^iv^	117.65 (13)	P2—O21—K1^xiii^	107.95 (16)
O24—Fe1—O24^iv^	84.56 (13)	Fe2^vi^—O21—K1^xiii^	105.53 (13)
O11—Fe1—O13	140.54 (13)	P2—O22—Fe2^ii^	138.67 (19)
O24—Fe1—O13	97.59 (13)	P2—O22—K1	106.73 (15)
O24^iv^—Fe1—O13	100.27 (13)	Fe2^ii^—O22—K1	111.49 (13)
O11—Fe1—O12	92.56 (13)	P2—O23—Fe2	139.32 (19)
O24—Fe1—O12	169.72 (13)	P2—O23—K1^i^	102.50 (14)
O24^iv^—Fe1—O12	95.47 (12)	Fe2—O23—K1^i^	113.17 (13)
O13—Fe1—O12	72.26 (12)	P2—O23—K1	96.07 (14)
O22^v^—Fe2—O21^vi^	87.21 (13)	Fe2—O23—K1	112.59 (13)
O22^v^—Fe2—O23	91.51 (13)	K1^i^—O23—K1	72.18 (8)
O21^vi^—Fe2—O23	91.82 (13)	P2—O24—Fe1	125.27 (18)
O22^v^—Fe2—O14	91.01 (13)	P2—O24—Fe1^iv^	130.84 (19)
O21^vi^—Fe2—O14	175.44 (13)	Fe1—O24—Fe1^iv^	95.44 (13)
O23—Fe2—O14	84.03 (13)	P2—O24—K1^i^	89.85 (13)
O22^v^—Fe2—O13^vi^	175.43 (13)	Fe1—O24—K1^i^	98.85 (12)
O21^vi^—Fe2—O13^vi^	93.14 (13)	Fe1^iv^—O24—K1^i^	111.87 (13)

Symmetry codes: (i) −*x*+1, −*y*, −*z*; (ii) −*x*+1/2, *y*−1/2, −*z*−1/2; (iii) *x*+1/2, −*y*−1/2, *z*+1/2; (iv) −*x*+1, −*y*, −*z*−1; (v) −*x*+1/2, *y*+1/2, −*z*−1/2; (vi) −*x*, −*y*, −*z*−1; (vii) *x*−1/2, −*y*+1/2, *z*+1/2; (viii) *x*, *y*, *z*−1; (ix) *x*−1/2, −*y*+1/2, *z*−1/2; (x) *x*+1/2, −*y*+1/2, *z*+1/2; (xi) *x*+1/2, −*y*+1/2, *z*−1/2; (xii) *x*, *y*, *z*+1; (xiii) *x*−1/2, −*y*−1/2, *z*−1/2.
